# Impact of Pre-Storage Melatonin Application on the Standard, Sensory, and Bioactive Quality of Early Sweet Cherry

**DOI:** 10.3390/foods12081723

**Published:** 2023-04-20

**Authors:** Daniel Cortés-Montaña, María Josefa Bernalte-García, Belén Velardo-Micharet, María Serrano, Manuel Joaquín Serradilla

**Affiliations:** 1 Department of Postharvest Science, Centre for Scientific and Technological Research of Extremadura, Avd. Adolfo Suárez s/n, 06007 Badajoz, Spain; daniel.cortes@juntaex.es (D.C.-M.); manuel.serradilla@juntaex.es (M.J.S.); 2 Department of Plant Biology, Ecology and Earth Sciences, University of Extremadura, Avd. Adolfo Suárez s/n, 06007 Badajoz, Spain; bernalte@unex.es; 3 Department of Applied Biology, University Miguel Hernández, Ctra. Beniel km. 3.2, 03312 Orihuela, Spain; m.serrano@umh.es

**Keywords:** *Prunus avium* L., elicitor, postharvest, sensory analysis, purchase intention, polyphenols

## Abstract

Melatonin (N-acetyl-5-methoxytryptamine) is involved in multiple functions in plants. However, its role in some metabolic pathways and exogenous application’s effect on fruits is still unclear. Furthermore, the effects of pre-storage melatonin treatment on sensory traits and consumer acceptance of cherries have yet to be studied. For this reason, the early sweet cherry cultivar ‘Samba’ harvested at the commercial ripening stage was treated with different melatonin concentrations (0.1, 0.3, and 0.5 mmol L^−1^) and stored for 21 days under controlled cold temperature and humidity. The standard quality, respiration rate, postharvest aptitude, sensory quality, phenols, and antioxidant systems (non-enzymatic and enzymatic) were analysed at 14 and 21 days of storage. Postharvest treatment with melatonin 0.5 mmol L^−1^ improved firmness and reduced weight loss and non-commercial fruit percentage while increasing respiration rate, lipophilic antioxidant activity, and ascorbate peroxidase enzyme activity. Furthermore, the treated cherries showed better sensory qualities, such as uniformity of colour and skin colour, as well as being sourer and showing better consumer acceptance and liking after 14 days of storage. Therefore, we conclude that the 0.5 mmol L^−1^ concentration is effective on the standard, sensory, and bioactive quality of early sweet cherries and can be considered an eco-friendly tool for maintaining the postharvest quality of early cherries.

## 1. Introduction

Turkey is the leading producer of cherry production, while Spain ranks sixth [1]. Spain exports only 13.3% of cherries outside the European Union, compared to 16.3% in 2019 [2]. In this context, exporting fruit to places further away from the point of production is becoming increasingly important. Sweet cherry (*Prunus avium* L.) is known to be a fruit highly appreciated in markets by consumers, not only for its organoleptic characteristics but also for its high antioxidant activity due to bioactive compounds, such as flavonoids, vitamins, and carotenoids [3,4,5]. However, cherries, especially early cultivars, have a very short shelf life of fewer than 15 days under low temperature and high relative humidity conditions [6]. Its high respiration rate, low carbon source content, and extreme sensitivity to mechanical damage or pitting cause significant economic and quality losses [4,5,7].

The high economic and nutritional value of cherries has encouraged many researchers to study their physiological and molecular basis of postharvest behaviour, particularly its impact on quality traits [3,8]. Postharvest technology has been mainly based, in the case of cherries, on the application of a passive modified atmosphere using plastic bags for export shipping [6]. However, the CO_2_ generated during storage in these bags is not enough to guarantee adequate quality at a long destination point [9]. For this reason, many researchers have focused on the search for safe and eco-friendly postharvest management or technology to delay the senescence and deterioration of cherries. Recently, studies have been carried out where biostimulators or elicitors have been used, such as methyl salicylate [10], methyl jasmonate [11], acetylsalicylic acid [12] and β-aminobutyric acid [13] that work as signalling compounds at low concentrations. These compounds act by triggering plant-induced resistance system response against biotic or abiotic stresses [14], delaying senescence and increasing the shelf life of sweet cherries.

On the other hand, many researchers have focused their studies on using melatonin (M) for its bioregulatory activity in plant physiological processes such as growth and development regulation, senescence delay and fruit ripening regulation. Melatonin (N-acetyl-5-methoxytryptamine) is a derivative of the amino acid tryptophan and was first identified in plants in 1995 [15]. In addition to acting as a signalling molecule against biotic and abiotic stresses, this molecule also plays an essential role as an antioxidant, scavenging reactive oxygen species and enhancing the role of other antioxidants [16,17,18]. Many works have shown that a melatonin treatment, depending on concentration and application time, can delay the senescence of fruit and vegetables and extend their commercial life, including sweet cherries [4,7,19,20,21,22]. 

Previous studies on postharvest melatonin dipping treatment in sweet cherries have been carried out by means of using applications at concentrations between 1 and 1000 µM at different temperatures, such as 5 and 20 °C, with a duration of 5 min [4,5,7,21]. In addition, these studies have been carried out using both early and mid-season cultivars such as ‘Santina’, ‘Sunburst’, ‘Ferrovia’, ‘Royal Rainer’ and ‘Siah Mashhad’. These previous studies have focused on understanding the response produced by the exogenous postharvest application of melatonin in cherries. They are mainly focused on the antioxidant mechanisms that regulate the dynamic equilibrium of reactive oxygen species (ROS) and the regulation of the genes involved, as well as their impact on physiological aspects of fruit, such as respiration rate or pedicel browning. However, to the best of our knowledge, none of the current studies has focused on the sensory attributes and consumers’ satisfaction with the product after storage. On the premise that exogenous melatonin applications improve fruit quality and shelf life by maintaining enzymatic and non-enzymatic antioxidant systems, in addition to determining the influence of different melatonin concentrations on the overall quality and antioxidant components of the fruit, the aim of this study also focused on the impact of these treatments on the sensory characteristics of early sweet cherries after cold storage.

## 2. Materials and Methods

### 2.1. Plant Material and Experimental Design 

The study was carried out with sweet cherries obtained from a commercial orchard of 25 eight-year-old trees of the early ‘Samba’ cultivar planted at 5 × 5 m and grafted onto Mariana-Adara rootstock, located in Jerte Valley (lat. 40°07′48.0″ N, long. 5°53′18.0″ W). Trees were subjected to standard fertiliser, herbicide, and pesticide practices. This early cultivar is firm and has a medium-sized stem, heart shape, dark red skin, and pale red flesh [23]. Sweet cherries were manually harvested in the early morning at commercial ripeness, stage 4 according to scale CTIFL, and transported at 5 °C to the laboratory within 2 h. About 60 kg of cherries homogeneous in colour, size and without visual defects were selected and randomly grouped in 4 batches of 15 kg each, which corresponds to control (CO) and three different melatonin concentrations 0.1 mmol L^−1^ (M0.1), 0.3 mmol L^−1^ (M0.3) and 0.5 mmol L^−1^ (M0.5). Hydrocooling treatment was applied until reaching a pulp temperature below 8 °C. Each batch was dipped in the different melatonin solutions (M0.1, M0.3 and M0.5) and CO in water for 10 min at 5.6 ± 0.5 °C and dried at room temperature for approximately 30 min. Subsequently, cherries were stored in 250 g macroperforated punnets at 1 ± 0.5 °C and 93.8 ± 2.7% relative humidity (RH) and analysed at 0, 14 and 21 days after a 4 h post-cooling period at room temperature (20 °C). All analyses were performed in triplicate and were shown as mean values ± standard error (SE).

### 2.2. Fruit Quality Parameters

Firmness was analysed by a 3% compression test on two opposite positions around the equator of 10 independent fruit for each replicate using a 25 mm flat base probe. A Stable Micro Systems TAXT2i texturometer (Aname, Pozuelo, Madrid, Spain) was used. The displacement speed of the test was 0.2 mm s^−1^, and the maximum force (N) was recorded from the force-deformation (N mm^−1^) curve. Skin and flesh colour were measured with a CR-4OO reflectance colourimeter (Minolta Camera Co, Osaka, Japan) with a D65 illuminant and an 8 mm diameter viewing area; CIELab space coordinates were recorded of 10 independent fruits for each replicate on four opposite sites around the equator for the skin and the two opposite sites for the flesh. The result was expressed as L* (luminosity) and a*/b* index, related to the fruit ripening stage [24]. With cherries used for firmness and colour measurement, 3 homogenates of 10 fruits each per treatment were prepared to measure total soluble solids (TSS) and titratable acidity (TA). TSS were measured at 20 °C with a portable digital refractometer PR-01 (Atago CO., LTD., Tokyo, Japan). Results were expressed as a percentage. For TA, 3 g of homogenate was diluted up to 60 mL with distilled water and titrated to pH 8.1 using 0.1 N NaOH in a Mettler Toledo DL50 titrator (Coslada, Madrid, Spain), with results expressed as % malic acid.

### 2.3. Respiration Rate

The respiration rate of whole sweet cherries was evaluated by a static system [25] using a PBI-Dansensor CheckMate 3 gas analyser (AMETEK INTRUMENTOS S.L., Barcelona, Spain). It was measured at 20 °C by placing each batch in a 1.7 L hermetic glass jar containing 250 g of sweet cherries (*n* = 3). Results were expressed as mg CO_2_ kg^−1^ s^−1^. 

### 2.4. Weight Loss and Disorder Evaluation

Weight loss was measured at each sampling point, compared with an initial weight of 250 g of sweet cherries (*n* = 3), and calculated in percentage [26]. Stem browning and non-commercial fruit were measured by visual inspection by qualified staff, as reported by Candan et al. [27]. Stem browning grading scale for stem browning was as follows: Grade 1: slight (less than 25% of the stem affected), Grade 2: moderate (between 25% and 50% of the stem affected), Grade 3: severe (between 50% and 75% of the stem affected) and Grade 4: very severe (more than 75% of the stem affected). Pitting is a term used to define cherries with severe mechanical damage. The result was expressed as a percentage of fruit showing this kind of damage.

### 2.5. Sensory Analysis and Consumer Satisfaction

A sensory panel of 12 previously trained panellists (6 males and 6 females aged between 30 and 50 years) conducted the sensory analysis on the 3 sampling points (0, 14 and 21 days), tasting 3 cherries each panellist per treatment (CO, M0.1, M0.3 and M0.5) after keeping them for 4 h at room temperature. Sensory properties related to appearance, colour, firmness, sweetness, sourness and other sensations were trained during 5 sessions. Informed consent was obtained from the panellists. Samples were randomly presented using three-digit reference codes. The sensory descriptors used were external appearance, colour uniformity, skin colour, sourness, sweetness, crispness, and firmness. A ten-point scale was used to judge the proposed descriptors. The sensory analysis was carried out in 12 individual standardised [28], using white illumination and at room temperature. Two tasting sessions were performed per sampling date.

A panel of fifty consumers (25 males and 25 females between 20 and 50 years) who were regular consumers of cherries randomly assessed 3 cherries from each treatment on each sampling date, also using a three-digit reference code and were asked to score their degree of disliking/liking using a nine-point hedonic scale (1, extreme dislike; 9, extreme like) for consumer satisfaction. After, consumers were asked about their purchase intention, answering yes or no based on all the sensory parameters previously evaluated.

### 2.6. Bioactive Compounds

At each sampling point, 15 fruit per treatment and replicate were frozen at −80°C. Independent homogenisations of 15 fruits (*n* = 3) from the different treatments were carried out to identify and quantify the bioactive compounds. 

#### 2.6.1. Identification and Quantification of Phenolic Compounds

Phenolic compounds were extracted as described in our previous work by Serradilla et al. [29]. Briefly, 10 g of homogenate was mixed with 50 mL of methanol (0.1% HCl), manually shaken and stored at −20 °C for 24 h. Separation and quantification were performed by HPLC-DAD/FLD, as described by Manzano et al. [30], using a Series 1100 chromatograph (Agilent) equipped with a degasser, a quaternary pump, a Gemini column (NX-C18, 150 × 4.6 mm, 3 µm) thermostated at 40 °C. Phenolic compounds were calculated using calibration curves that were made with standards and following the method of external standards. Results were expressed as g kg^−1^ of fresh weight (FW).

#### 2.6.2. Antioxidant Activity

Total antioxidant activity (TAA), both hydrophilic and lipophilic fractions (H-TAA and L-TAA), was measured according to Serrano et al. [31]. For each sample, 5 g of homogenate was mixed with 10 mL of phosphate buffer (pH 7.5, 50 mM) and 3 mL of ethyl acetate. It was centrifuged at 15,071× *g* for 30 min at 4 °C. The upper (lipophilic) phase was used to determine L-TAA and the lower (hydrophilic) phase for H-TAA. In both cases, TAA was determined using the enzymatic system consisting of ABTS, the enzyme peroxidase and its substrate (hydrogen peroxide). Results were expressed as g Trolox equivalent Kg^−1^ of fresh weight (FW). 

#### 2.6.3. Enzymatic Antioxidant Activity

The activity of the enzymes peroxidase (POD), catalase (CAT) and ascorbate peroxidase (APX) was measured as described by Carrión-Antoli et al. [32]. Briefly, 5 g homogenate was mixed with 10 mL of 50 mM phosphate buffer, pH = 7.0, containing 1% (*p/v*) polyvinylpyrrolidone and 1 mM ethylenediaminetetraacetic acid. It was centrifuged at 21,100× *g* for 30 min at 4 °C, and the supernatant was used for enzyme assays. Results were expressed as U min^−1^ g protein^−1^, with one enzymatic unit (U) being a 0.01 absorbance increase per min.

### 2.7. Statistical Analysis 

Experimental data were subjected to one-way and two-way ANOVA analysis using SPSS Statistics 25.0 (IBM, NY, USA) software for Windows. The significant effect of treatment and storage time on the variables under study was evaluated at a *p*-value ≤ 0.05. Multiple comparisons test was carried out using Tukey’s HSD post hoc test. 

## 3. Results

### 3.1. Quality Parameters

**Figure 1:**
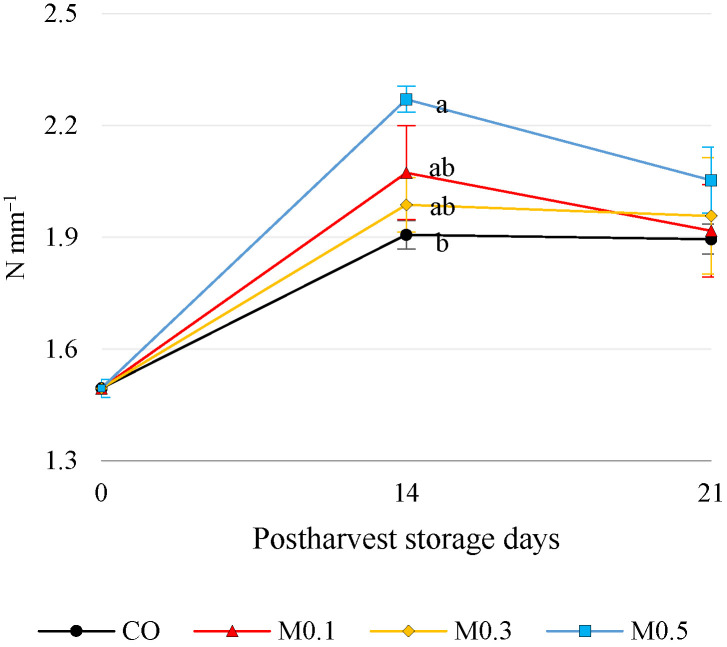
Fruit firmness (N mm^−1^). Mean values ± SE (*n* = 30). By storage time, different letters indicate significant differences among treatments by Tukey’s test (*p* ≤ 0.05).

In this current study, fruit firmness at harvest (Day 0) was 1.50 ± 0.03 N mm^−1^ (Figure 1). 

On the one hand, treated and untreated ‘Samba’ cultivar cherries exhibited a significant increase (*p* ≤ 0.05) in firmness during cold storage, reaching maximum firmness values after 14 days of cold storage for all treatments (Figure 1). On the other hand, melatonin-treated fruit provided higher values than untreated fruit throughout storage, with M0.5 treated cherries (2.27 ± 0.04 N/mm) showing significant differences (*p* ≤ 0.05) with untreated cherries (1.91 ± 0.04 N/mm) after 14 days.

With regard to objective colour, both skin and flesh colour showed no significant differences between untreated and treated sweet cherries throughout storage, but there were among dates for L* skin and a*/b* flesh (Table 1). 

L* skin values ranged from 28.24 ± 0.11 at harvest to 29.98 ± 0.12 after 21 days of storage, while flesh a/b ratio ranged from 1.83 ± 0.03 (Day 0) to 2.03 ± 0.02 after 21 days of storage. It is also remarkable that flesh a/b was the only colour parameter that showed a significant interaction between sampling day and treatment (Table 1). TSS content ranged from 15.73 ± 0.19% to 14.94 ± 0.15% throughout cold storage, showing significant differences (*p* ≤ 0.05) among sampling points, but no differences were observed among treatments at the same sampling point. However, after 21 days of cold storage, TSS content was significantly lower than at harvest and 14 days (Table 1). In this line, TA also decreased during storage, but no significant differences were observed among treatments. The initial value was 0.88 ± 0.01% malic acid, and it decreased significantly (*p* ≤ 0.05) at 14 and 21 days, reaching 0.63 ± 0.01% malic acid after 21 days of storage.

### 3.2. Respiration Rate

**Figure 2:**
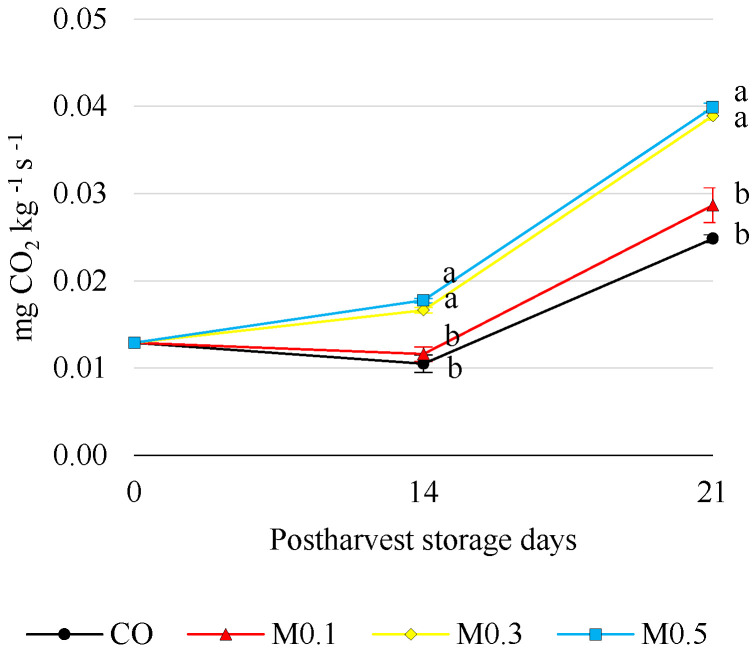
Respiration rate (mg CO_2_ kg^−1^ s^−1^) of ‘Samba’ cherries in relation to treatments and storage time. Mean values ± SE *(n* = 3). By storage time, different letters indicate significant differences among treatments by Tukey’s test (*p* ≤ 0.05).

The respiration rate at the time of harvest was 0.01 ± 0.00 mg CO_2_ kg^−1^ s^−1^, while during cold storage, significant differences (*p* ≤ 0.05) were observed between CO and M0.1 with M0.3 and M0.5, the latter two treatments showing the highest respiration rates and a significant increase after 14 and 21 days of cold storage (Figure 2). 

### 3.3. Weight Loss and Disorder Evaluation

**Figure 3:**
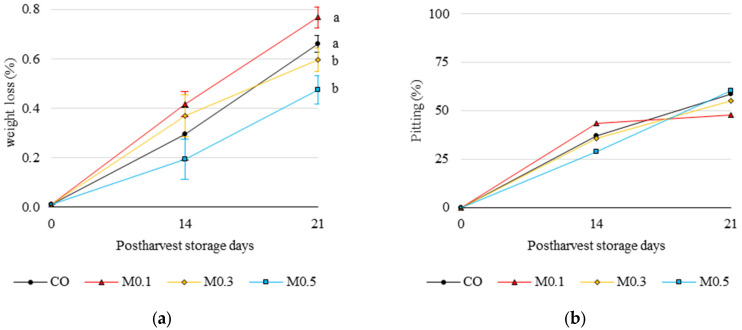
(**a**) Weight loss (%) of ‘Samba’ cherries in relation to treatments and storage time. (**b**) Pitting (%) of ‘Samba’ cherries in relation to treatments and storage time. Mean values ± SE (*n* = 3). By storage time, different letters indicate significant differences between treatments by Tukey’s test (*p* ≤ 0.05).

Weight loss increased throughout storage, with CO and M0.1 showing a significant steady weight loss, with 0.30 ± 0.17% and 0.42 ± 0.05% at 14 days and 0.66 ± 0.04% and 0.77 ± 0.04% at 21 days of storage, respectively (Figure 3a). After 21 days of storage, weight loss of M0.3 and M0.5 treated cherries was significantly lower (*p* ≤ 0.05) with M0.1 and CO.

Concerning stem browning, its severity remained at grade 1 up to 21 days of storage, and there were no significant differences for this disorder. However, there was a high percentage of pitting (severe mechanical damage) during cold storage (Figure 3b), ranging from 36% to 55% at 14 and 21 days, respectively. In general, treated cherries showed the lowest percentages of non-commercial fruit.

### 3.4. Sensory Analysis and Consumer Satisfaction

Significant differences (*p* ≤ 0.05) were observed in all sensory attributes tested, except crispness and firmness. The external appearance was very similar for all treatments, scoring very high (8.6) at the beginning and significantly lower at 14 and 21 days of storage (6.9 and 6.5, respectively). Colour uniformity was generally fairly stable throughout cold storage, with significant differences (*p* ≤ 0.05) among treatments at 14 days, with M0.3 and M0.5 treatments showing significantly higher values than untreated cherries (Table 2).

Skin colour was evaluated visually by each panellist according to a scale from 1 (light red) to 10 (black). All cherries showed a mahogany colour (7.2) at the beginning of the trial. The colour remained fairly stable throughout storage in all treatments, except for M0.3 cherries, which acquired a darker colour (8.4) at the end of the trial. At 21 days, in addition, significant differences (*p* ≤ 0.05) were observed among treatments, with M0.1 treated fruit showing a lower skin colour than M0.3 (Table 2). 

Sweet cherries received an initial sensory score of 5.2 for sourness independently of treatment, which corresponds to sour cherries (0.6–0.7% of TA) according to the training previously carried out for this attribute. A significant decrease in sourness was observed after 14 days of cold storage in CO and M0.3 treatments, remaining stable in M0.1 and M0.5 cherries. By treatments, significant differences (*p* ≤ 0.05) were only found at 14 days of storage, with M0.5 samples showing significantly higher values than CO (Table 2). At the end of storage, sweet cherry sourness decreased up to 3.9, included in the range of low-acid fruits (0.4–0.6% of TA).

The sweetness was, overall, fairly stable throughout storage at 2 °C (Table 2), with ‘Samba’ cherries scoring an average of 4.6 ± 0.4 for this attribute. This value corresponds to a description of slightly sweet cherries (16–18 °Brix). By treatments, significant differences (*p* ≤ 0.05) were only found at 14 days of storage, with M0.1 significantly lower than CO.

The degree of disliking/liking was evaluated using a nine-point hedonic scale. Although no significant differences were found either among treatments or among sampling points, an evolution in consumer satisfaction with cherries was observed (Table 3). The initial fruit (day 0) obtained a high score in consumer satisfaction, with 81.8% of scores being equal to or higher than “I like it slightly” to “Think it’s wonderful”. After 14 days of cold storage, it decreased, with 75% of scores similar to or higher than “I like it slightly”. Treatments M0.1 and M0.5 showed 75% above the level “I like it slightly”, and M0.3 maintained a high percentage of consumer satisfaction (83.4% of scores equal to or higher than “I like it slightly”), while the other treatments began to show indifferent scores (I neither like nor dislike it), with an average of 16.7%. Finally, at the end of storage, consumer satisfaction with cherries continued to decline, and the percentage of indifference and dissatisfaction scores increased. In general, M0.3 and M0.5 showed the highest level of consumer satisfaction throughout cold storage, mainly at 14 days (which is the maximum recommended cold storage for this cultivar), while M0.1 treated cherries scored less than or equal to CO.

Purchase intention represents the percentage of consumers who, after tasting the fruit, answered that they would repurchase it. According to this parameter, no significant differences were found either among treatments or sampling dates. Nevertheless, the purchase intention evolved positively from day 0 (72.7%) to day 14 (85.4%), decreasing after 21 days of storage at 64.2%. By treatments, at 14 days, all melatonin treatments received a higher purchase intention than CO (75%), especially M0.5 (91.7%) and M0.3 (91.7%). Finally, after 21 days of cold storage, purchase intention drops to 75% for M0.5, 66.7% for M0.3, 65% for CO and 50% for M0.1.

### 3.5. Bioactive Quality

#### 3.5.1. Identification and Quantification of Phenolic Compounds 

The phenolic compounds identified and quantified in the early Samba cultivar were grouped into anthocyanins, hydroxycinnamic acids, flavonols and flavan-3-ols (Table 4).

For all phenolic compounds observed, a decrease in concentration could be observed throughout storage, although in the case of melatonin-treated cherries, especially in M0.5-treated cherries, this loss was lower, except for flavonols. Within the anthocyanins, the major phenolic compounds responsible for their characteristic colour, the concentration of the cyanidin 3-*O-*rutinoside, showed significant differences (*p* ≤ 0.05) between treated and untreated cherries. Melatonin-treated cherries maintained higher concentrations of this compound throughout storage and, with it, a better skin colour. On the other hand, significant differences (*p* ≤ 0.05) were also found in the total sum of anthocyanins, with cherries treated with melatonin showing the highest concentration. It should be noted that a strong interaction (*p* ≤ 0.001) was observed between the two study factors, storage time and treatment on the total anthocyanin concentration (Table 4), evidencing that postharvest treatment with melatonin has a positive effect on anthocyanins. 

Another group of phenolics of high importance in cherries are the hydroxycinnamic acids, especially the neochlorogenic acid and *p*-coumaroylquinic acid content. Both hydroxycinnamic acids were higher in the M0.5 melatonin-treated cherries, with significant differences (*p* ≤ 0.001) with untreated cherries in the case of neochlorogenic acid. As with anthocyanins, the total sum of hydroxycinnamic acids was significantly influenced (*p* ≤ 0.05) by the interaction of treatment and storage time, showing a positive effect of postharvest treatment with melatonin on this group of polyphenols.

In the case of flavonols, significant differences (*p* ≤ 0.05) were only found between cherries treated with M0.3 and untreated cherries in the concentration of kaempherol 3-*O*-rutinoside.

Regarding flavan-3-ols, significant differences (*p* ≤ 0.05) were found between M0.5 melatonin-treated and untreated cherries for the concentration of procyanidin PB2, (+)-Catechin and (−)-epicatechin. In general, postharvest treatment with melatonin had a positive effect in delaying the loss of polyphenols, except flavonols, throughout the storage process and, thus, preserving the bioactive characteristics of this fruit. 

#### 3.5.2. Antioxidant Activity

**Figure 4:**
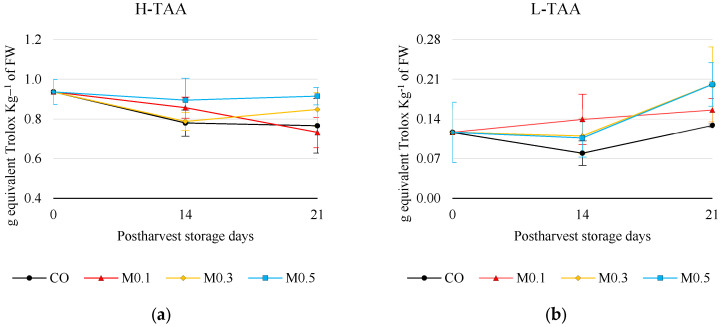
Antioxidant activity of ‘Samba’ cherries in relation to treatments and storage time. (**a**) Hydrophilic total antioxidant activity (H-TAA). (**b**) Lipophilic total antioxidant activity (L-TAA). Mean values ± SE (*n* = 3).

Hydrophilic total antioxidant activity (H-TAA) was not influenced by pre-storage melatonin application (Figure 4a). The mean results were 0.94 ± 0.06 g equivalent Trolox Kg^−1^ of FW at 0 days and 0.83 ± 0.07 and 0.82 ± 0.09 (g Kg^−1^) at 14 and 21 days of cold storage, respectively (Figure 4a). In the case of M0.1-treated cherries, it was the only treatment that suffered a significant decrease (*p* ≤ 0.05) throughout cold storage. However, M0.3 and M0.5 treated cherries showed a better H-TAA than CO, highlighting that M0.5 treated cherries showed an H-TAA level more like day 0. By contrast, melatonin treatments induced a sharp increase of 33.62% (M0.1), 72.67% (M0.3), and 72.45% (M0.5) in lipophilic total antioxidant activity (L-TAA) at 21 days of storage compared to day 0 and untreated cherries (Figure 4b).

#### 3.5.3. Enzymatic Antioxidant Activity

**Figure 5:**
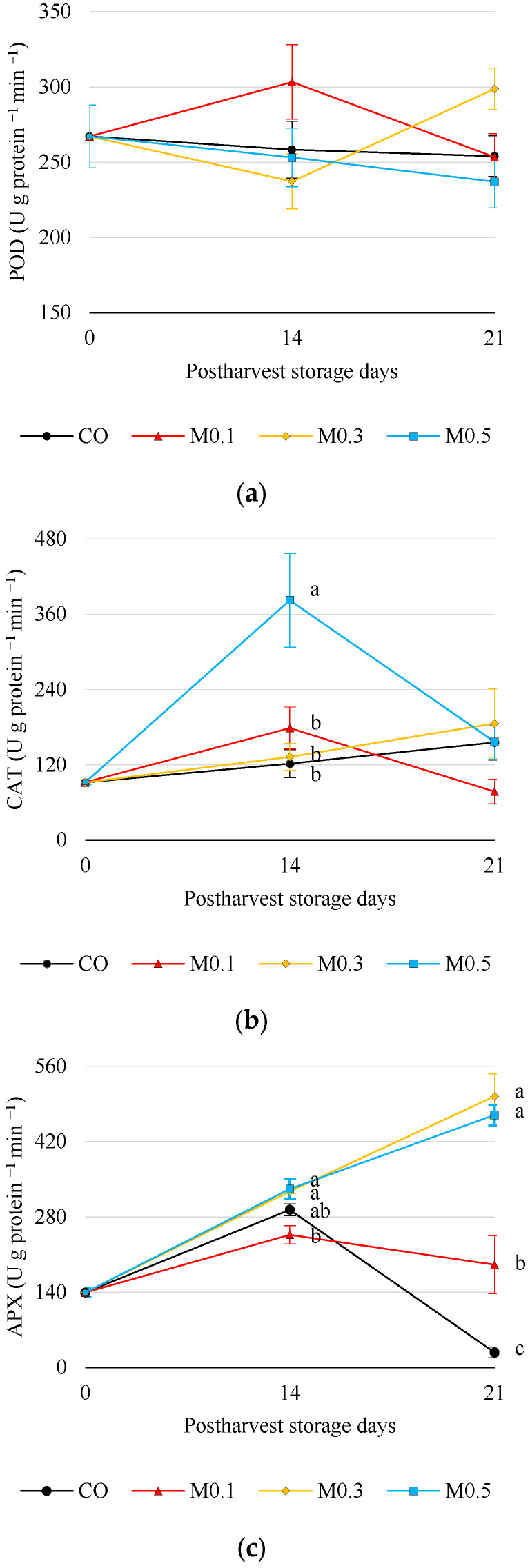
Enzymatic antioxidant activities of ‘Samba’ cherries in relation to treatments and storage time. (**a**) Peroxidase antioxidant activity (POD) (U g protein ^−1^ min ^−1^), (**b**) Catalase antioxidant activity (CAT) (U g protein ^−1^ min ^−1^), (**c**) Ascorbate peroxidase antioxidant activity (APX) (U g protein ^−1^ min ^−1^). Mean values ± SE. By storage time, letters indicate significant differences between treatments at *p* ≤ 0.05 (Tukey’s test).

The activity of antioxidant enzymes peroxidase (POD), catalase (CAT) and ascorbate peroxides (APX) was measured throughout cold storage. The antioxidant enzyme POD showed steady activity throughout storage (Figure 5a), showing a mean value of 263.55 ± 18.59 (U min^−1^ g protein^−1^). Because of that, there were no significant differences between treated and untreated cherries, although it was observed that M0.3 treated cherries showed the highest activity for this antioxidant enzyme after 21 days of storage. With regard to CAT (Figure 5b), it was observed that M0.5 treated cherries showed a higher value of 382.25 ± 74.76 (U min^−1^ g protein^−1^) after 14 days of cold storage and significant differences (*p* ≤ 0.05) compared to both other treatments and CO that showed a mean value of 289.11 ± 10.71 U min^−1^ g protein^−1^. A growing trend was observed in APX activity during the first 14 days of postharvest storage (Figure 5c). However, a great impact of exogenous pre-storage M0.3 and M0.5 treatments showed a significant increase in APX activity, thereafter 21 days of storage, especially in control fruit.

## 4. Discussion

In recent literature, there are several works on the effect of postharvest melatonin dipping in sweet cherries, where melatonin is not only a safe and non-toxic substance for humans but also an important signalling molecule that functions as a master regulator of certain physiological traits and antioxidant systems of fruits, delaying postharvest diseases and senescence process in sweet cherries [4,5,7,21,33,34]. Nevertheless, little is known about the effect of exogenous postharvest melatonin application on sensory traits and consumer acceptance. 

Sweet cherry quality is evaluated through its physicochemical parameters and evolution throughout its commercial life or shelf life. Losses in weight, firmness and titratable acidity during storage and subsequent shipment, along with changes in skin colour and total soluble solids content, define their quality and consumer acceptance as well as their repurchase [32]. Once harvested, they begin to undergo physiological changes that cause rapid deterioration of the fruit, and consequently, cherries that reach consumers may not have the optimal organoleptic characteristics [6,8]. In the present study, the early cultivar ‘Samba’ was used, and as in the other early sweet cherry cultivars, its postharvest life is short if no postharvest technologies are used [6]. 

Firmness is a key factor determining sweet cherry shelf life [6]. The firmness of melatonin-treated fruit was higher than in untreated cherries, with significant differences (*p* ≤ 0.05) only at M0.5 (Figure 1). This effect of melatonin on firmness during storage was reported by [32] in cultivars Prime Giant and Sweetheart using these same concentrations in the preharvest application. However, Carrión-Antolí et al. and Bal et al. [4,33] found that both melatonin-treated and untreated cherries decreased in firmness during storage, although this decline in firmness was delayed in melatonin-treated cherries. This finding highlights the need to adjust melatonin concentration for each cultivar. Melatonin is an agent involved in multiple physiological processes and has excellent antioxidant potential as a free radical scavenger [34]. Its amphiphilic nature allows it to distribute throughout the cell freely; it can cross cell membranes and be found in the cytosol, mitochondria or nucleus, fulfilling protective functions against reactive nitrogen and oxygen species (ROS) [16,35,36,37]. Accordingly, Wang et al. [4] and Bal et al. [33] showed that during sweet cherry postharvest, the senescence process is accelerated, and this oxidative process is associated with the accumulation of ROS along with overactivity of the membrane-degrading enzymes phospholipase D (PLD) and lipoxygenase (LOX). This aspect favours the peroxidation of membrane lipids that promotes a loss of internal structures and, consequently, a decrease in fruit firmness. However, as mentioned above, exogenous melatonin application helps regulate intracellular ROS accumulation, thus maintaining cell integrity [4,5]. 

‘Samba’ sweet cherry is classified as a fruit with a high respiration rate as compared with other cultivars [38,39]. It is essential to control this metabolic activity to increase shelf life. In the application of exogenous melatonin, there is much controversy regarding its effects on this parameter. On the one hand, Wang et al. [4], Miranda et al. [21] and Bal et al. [33] reported that exogenous melatonin applications to sweet cherries resulted in delayed respiration leading to reduced weight loss and delayed senescence. In contrast, authors such as Michailidis et al. [7] and Fan et al. [40] found an increase in the respiration rate of fruit treated with melatonin. It was observed in ‘Ferrovia’ sweet cherry that melatonin applications, especially the preharvest and postharvest combination, increased respiration during storage at low temperatures [7]. According to these authors, the exogenous application of melatonin and cold storage triggers the activation of genes related to the tricarboxylic acid (TCA) cycle. Based on our results, an increase in respiration activity was observed in melatonin-treated cherries of the Samba cultivar with respect to CO. Moreover, this increase was proportional to the dose applied, with M0.5-treated cherries showing the highest respiration rate throughout storage. This fact could lead to an intracellular unbalance in ROS accumulation [4], leading to a higher weight loss due to oxidations of the cellular structure. However, it should be noted that the lowest weight loss throughout storage was found in cherries treated with a 0.5 mM exogenous dose of melatonin (Figure 2). This aspect needs to be studied in more depth in future research.

Postharvest melatonin treatments led to a lower incidence of quality loss during cold storage. Thus, in our study, after 14 and 21 days of cold storage in macroperforated punnets, the percentage of non-commercial fruit exceeded 30% and 50%, respectively. No significant differences were found among treatments, but it could be observed that M0.3 and M0.5 treated cherries showed lower percentages than CO after the first sampling date. Previous studies also found a lower percentage of decay incidence in melatonin-treated fruit [4,5]. Thus, considering this parameter, the optimal storage time for this cultivar under these conditions would be 14 days; this finding is in accordance with those obtained by Zoffoli et al. [6]. 

Concerning weight losses, M0.3 and M0.5 treated cherries showed significantly lower weight losses at the end of storage (Figure 3a). These results agree with Miranda et al. [21], who also observed the same trends in ‘Santina’ and ‘Royal Lee’ early cultivars after 21 days of storage. According to these authors, melatonin activates the accumulation of abscisic acid and, in turn, also regulates the transport of water across the membrane and cuticle. Nije et al. [41] and Lin et al. [42] also observed maintenance of firmness and weight in the treated fruit due to melatonin-promoted synthesis of long-chain alkanes that were positively correlated with water retention and firmness.

Fruit flavour and consumer acceptance are closely related to TSS and TA parameters [8,43]. According to Nije et al. [41], in our study, there were no significant differences among treatments in these parameters, but there was a significant decrease (*p* ≤ 0.05) in TSS and TA throughout storage (Table 1). It should be noted that the cherries treated with melatonin showed lower TA values than the control because these cherries showed a higher respiratory rate. It is well known that organic acids act as a substrate for the tricarboxylic acid cycle [14]. These findings are consistent with those obtained by Bal et al. [33] on the Sweetheart late cultivar. Nevertheless, Wang et al. and Miranda et al. found an increase in TSS and TA in cherries treated with exogenous melatonin in different sweet cherry cultivars [4,21]. This result is further evidence that the effect of exogenous melatonin is genotype-dependent and dose-dependent. 

On the other hand, in this study, trained panellists found differences in sweetness and sourness after 14 days of storage (Table 2), with melatonin-treated cherries showing a lower degree of sensory ripeness and a delay in senescence, despite having shown an increase in the respiration rate. Several authors have reported that the application of melatonin, both preharvest and postharvest, leads to a delay in ripening and senescence during storage [7,32,44]. In a study carried out with the early cultivar ‘Early Bigi’ by López et al. [8], these authors concluded that sweet cherry flavour was directly related to fruit acidity. In our study, trained panellists identified the melatonin-treated cherries with the highest sourness scores, especially those of M0.3 and M0.5, despite showing no difference in titratable acidity. Furthermore, these cherries also showed the best consumer acceptance. Therefore, these sensory analysis results also show that acidity is critical to the flavour and acceptability of the fruit.

Among the parameters that most influence the decision to purchase cherries is colour [24]. In addition, skin colour darkening, as a marker of senescence, is a primary factor restricting the quality and shelf life of cherries during marketability. The sensory analysis of skin colour and uniformity revealed that trained panellists rated the M0.3 and M0.5 treated cherries with the highest scores. This improvement in skin colour from a sensory point of view and the delay of senescence may explain why the treated cherries showed better acceptability values and better consumer acceptance after 14 days of storage. 

During this study, it was found that melatonin enhances the enzymatic and non-enzymatic antioxidant systems of the ‘Samba’ sweet cherry. These results agree with other authors’ results in sweet cherry [4,7,21,32,34]. Regarding non-enzymatic systems, exogenous melatonin treatments improved both hydrophilic and lipophilic fruit antioxidant activity (Figure 4), although no increase in phenolic compound content was observed (Table 4). Most of the antioxidant activity of sweet cherries is due to their high flavonoid content, especially anthocyanins [3]. Liu et al. reported that the accumulation of phenolic compounds was linked to increased ABTS^-+^ radical scavenging potential and suppression of endogenous H_2_O_2_ accumulation [20]. Different authors [4,7,21,34] reported that melatonin induces the synthesis and accumulation of phenolic compounds, mainly anthocyanins. This effect was due to melatonin activation of genes involved in the phenolic compound synthesis pathway during cold storage, such as, among others, 4-coumarate (Pa4CL), cinnamate-4-hydroxylase (PaC4H) and dihydroflavonol 4-reductase (PaDFR) [7]. It was observed that postharvest application of melatonin at the maximum concentration of 0.5 mM maintained significantly higher levels of anthocyanins, hydroxycinnamic acids and flavan-3-ols than untreated cherries during storage (Table 4). This positive effect of melatonin was not found for flavonols. The maintenance of these flavonoids would also explain why M0.5-treated cherries presented better sensory characteristics, better consumer satisfaction and better purchase intention after 14 days of storage.

Regarding enzymatic antioxidant activity, melatonin postharvest application to the cultivar ‘Samba’ increased POD and CAT activity at day 14 and APX activity at 21 with respect to the control fruit (Figure 5). This led to improved firmness (Figure 1) and reduced pitting expression (Figure 3b). Wang et al. [4] also observed the effect of melatonin on antioxidant enzyme activity (CAT and APX) in the ‘Sunburst’ sweet cherry cultivar. As mentioned above, this stimulation of CAT and APX enzyme activity maintains membrane integrity due to the neutralisation of ROS, limitation of lipoxygenase (LOX) and PPO activity and reduction of malondialdehyde accumulation [4,19,32,41,45]. Therefore, the exogenous application of melatonin enhances the ROS scavenging systems (enzymatic and non-enzymatic), which would lead to the maintenance of cellular structures and thereby delay senescence and increase shelf life. 

## 5. Conclusions

Pre-storage application of melatonin, especially 0.5 mmol L^−1^, by immersion, maintained the enzymatic and non-enzymatic antioxidant systems of ‘Samba’ sweet cherry during storage, providing firmer cherries without negatively altering skin colour and flavour, leading to improved organoleptic characteristics as well as better purchase intention after 14 days of storage. Therefore, applying melatonin before storage can be considered an excellent postharvest tool to maintain the standard, sensory, and bioactive quality of early cherries. However, more studies are needed to clarify the mechanism of action of melatonin.

## Figures and Tables

**Table 1:** Mean values ± SE of skin and flesh colour, total soluble solids (TSS), and titratable acidity (TA) of ‘Samba’ cherries in relation to storage time and treatments.

	L* Skin	a*/b* Skin	L* Flesh	a*/b* Flesh	TSS (%)	TA (%)
Storage time						
0	28.24 ± 0.11 ^b1^	3.92 ± 0.09	42.16 ± 0.71	1.83 ± 0.03 ^b^	15.73 ± 0.19 ^a^	0.88 ± 0.01 ^a^
14	28.69 ± 0.17 ^b^	3.77 ± 0.06	42.23 ± 1.03	1.84 ± 0.06 ^b^	15.83 ± 0.23 ^a^	0.61 ± 0.04 ^b^
21	29.98 ± 0.12 ^a^	3.72 ± 0.05	40.16 ± 0.50	2.03 ± 0.02 ^a^	14.94 ± 0.15 ^b^	0.63 ± 0.01 ^b^
Treatment						
CO	28.77 ± 0.31	3.85 ± 0.77	40.29 ± 0.91	1.98 ± 0.05	15.61 ± 0.29	0.73 ± 0.04
M0.1	28.96 ± 0.33	3.82 ± 0.09	41.74 ± 0.86	1.89 ± 0.05	15.54 ± 0.88	0.72 ± 0.05
M0.3	29.16 ± 0.30	3.77 ± 0.09	42.30 ± 0.74	1.85 ± 0.04	15.32 ± 0.64	0.71 ± 0.04
M0.5	29.00 ± 0.27	3.77 ± 0.09	41.74 ± 1.18	1.87 ± 0.08	15.52 ± 0.78	0.66 ± 0.06
*p* date ^2^	***	ns	ns	***	*	***
*p* treatment	ns	ns	ns	ns	ns	ns
*p* date * treatment	ns	ns	ns	*	ns	ns

Data were presented as a mean value of 12 replicates for storage time and 9 for treatment. Mean values ± SE. ^1^ In each column, different letters indicate a significant difference at *p* ≤ 0.05 (Tukey’s test). ^2^ *p* values: *** (*p* ≤ 0.001); * (*p* ≤ 0.05); ns: not significant.

**Table 2:** Mean scores obtained in the sensory evaluation of ‘Samba’ cherries in relation to treatments and storage time.

Sensory Attributes	0 Days	14 Days				21 Days			
	CO	CO	M0.1	M0.3	M0.5	CO	M0.1	M0.3	M0.5
Colour uniformity	8.6	7.5 ^b^*	7.7 ^ab^	8.5 ^a^	8.5 ^a^	8.8	8.2	9.1	9.2
Skin colour	7.2	7.0	6.6	7.6	7.6	7.4 ^ab^	7.2 ^b^	8.4 ^a^	7.8 ^ab^
Sourness flavour	5.2	3.9 ^b^	4.8 ^ab^	4.0 ^b^	5.0 ^a^	3.7	3.8	3.9	4.3
Sweetness flavour	4.6	5.6 ^a^	4.1 ^b^	4.7 ^ab^	4.5 ^ab^	3.8	4.2	4.0	4.1

* By storage time, different letters indicate significant differences between treatments by Tukey’s test (*p* ≤ 0.05).

**Table 3:** Degree of disliking/liking (%) and purchase intention (%) of ‘Samba’ cherries in relation to storage time and treatments.

Consumer Satisfaction	0 Days	14 Days				21 Days			
	CO	CO	M0.1	M0.3	M0.5	CO	M0.1	M0.3	M0.5
I quite dislike it	0	0	0	0	0	0	0	8.3	0
I dislike it slightly	18.2	16.7	8.3	8.3	0	8.3	8.3	8.3	16.7
I neither like it nor dislike it	0	16.7	16.7	8.3	25	25	50	16.7	8.3
I like it slightly	18.2	25	58.3	50	50	50	16.7	50	41.7
I quite like it	36.3	16.7	16.7	16.7	0	16.7	16.7	8.3	8.3
I like it very much	18.2	25	0	16.7	25	0	8.3	8.3	8.3
Think it’s wonderful	9.1	0	0	0	0	0	0	0	0
Purchase intention	72.7	75	83.3	91.7	91.7	65	50	66.7	75

**Table 4:** Identification and quantification of phenolic compounds of ‘Samba’ sweet cherry in relation to storage time and treatments. Mean values 10^−2^ (g k^−1^) ± SE.

Polyphenols		Storage Time				Treatment					
		0	14	21	*p* ^ #^	CO	M0.1	M0.3	M0.5	*p* ^ ##^	*p* ^ ###^
Anthocyanins	C3G	2.23 ± 0.05 ^a1^	1.04 ± 0.25 ^b^	1.03 ± 0.29 ^b^	ns	1.03 ± 0.32	0.98 ± 0.36	0.88 ± 0.96	1.06 ± 0.33	ns	ns
	C3R	37.67 ± 3.78 ^a^	26.26 ± 3.47 ^b^	26.05 ± 5.97 ^b^	ns	23.21 ± 4.68 ^b^	29.53 ± 8.32 ^a^	23.62 ± 2.78 ^ab^	24.49 ± 4.68 ^ab^	*	***
	P3G	1.83 ± 0.22	1.35 ± 0.23	1.35 ± 0.44	ns	1.35 ± 0.36	1.40 ± 0.22	1.38 ± 0.32	1.26 ± 0.15	ns	ns
	P3R	0.10 ± 0.17	0.19 ± 0.02	0.35 ± 0.51	ns	0.19 ± 0.01	0.19 ± 0.03	0.49 ± 0.72	0.20 ± 0.02	ns	ns
	SAN	41.84 ± 3.75 ^a^	28.99 ± 4.42 ^b^	28.63 ± 6.58 ^b^	ns	25.78 ± 5.20 ^b^	32.21 ± 6.88 ^a^	27.20 ± 2.05 ^ab^	30.03 ± 1.68 ^ab^	*	***
Hydroxycinna mic	NEO	32.80 ± 3.11 ^a^	27.40 ± 2.39 ^b^	26.44 ± 3.53 ^b^	ns	24.87 ± 2.99 ^b^	25.69 ± 1.84 ^b^	26.71 ± 1.61 ^ab^	30.40 ± 2.17 ^a^	***	*
	*PCQ*	9.39 ± 1.68	9.32 ± 1.46	9.37 ± 1.38	ns	8.31 ± 1.05	9.44 ± 1.26	9.33 ± 0.97	10.32 ± 1.68	ns	ns
	CHL	2.48 ± 0.35 ^a^	2.20 ± 0.27 ^a^	1.80 ± 0.23 ^b^	****	1.87 ± 0.42 ^b^	1.93 ± 0.27 ^b^	2.06 ± 0.40 ^a^	2.13 ± 0.15 ^a^	ns	*
	*PCO*	0.00 ± 0.00	0.07 ± 0.07	0.03 ± 0.07	ns	0.04 ± 0.05	0.07 ± 0.08	0.03 ± 0.05	0.07 ± 0.08	ns	+
	SHA	44.66 ± 4.89 ^a^	38.99 ± 3.73 ^b^	37.65 ± 4.88 ^b^	ns	35.08 ± 4.37 ^b^	37.14 ± 2.58 ^ab^	38.13 ± 2.32 ^ab^	42.92 ± 3.80 ^a^	**	*
Flavonols	Q3R	2.92 ± 0.35 ^a^	1.98 ± 0.35 ^b^	1.99 ± 0.20 ^b^	ns	2.02 ± 0.16	1.93 ± 0.37	1.95 ± 0.18	1.72 ± 0.27	+	*
	K3R	0.58 ± 0.12 ^a^	0.28 ± 0.12 ^b^	0.28 ± 0.07 ^b^	ns	0.22 ± 0.05 ^b^	0.46 ± 0.13 ^b^	0.49 ± 0.06 ^a^	0.45 ± 0.08 ^b^	+	+
	I3R	0.60 ± 0.13	0.47 ± 0.06	0.77 ± 1.06	ns	1.10 ± 1.48	0.46 ± 0.10	0.47 ± 0.06	0.42 ± 0.06	ns	ns
	SFL	4.18 ± 0.64 ^a^	2.49 ± 0.38 ^b^	3.07 ± 1.12 ^ab^	ns	3.16 ± 1.65 ^a^	2.95 ± 0.40 ^a^	2.79 ± 0.19 ^a^	2.66 ± 0.34 ^b^	ns	ns
Flavan-3-ols	PB1	1.71 ± 0.15 ^a^	1.39 ± 0.14 ^b^	1.38 ± 0.14 ^b^	ns	1.29 ± 0.10	1.49 ± 0.07	1.32 ± 0.17	1.45 ± 0.07	ns	ns
	PB2	0.88 ± 0.18 ^a^	0.48 ± 0.09 ^b^	0.49 ± 0.15 ^b^	ns	0.45 ± 0.08 ^b^	0.41 ± 0.11 ^b^	0.45 ± 0.07 ^b^	0.63 ± 0.06 ^a^	*	ns
	CAT	0.85 ± 0.16 ^a^	0.59 ± 0.08 ^b^	0.62 ± 0.12 ^b^	ns	0.55 ± 0.04 ^b^	0.59 ± 0.07 ^ab^	0.59 ± 0.09 ^ab^	0.65 ± 0.14 ^a^	ns	ns
	EPI	4.29 ± 1.35 ^a^	2.97 ± 0.54 ^b^	3.24 ± 0.85 ^b^	ns	2.64 ± 0.30 ^b^	2.84 ± 0.50 ^ab^	3.00 ± 0.32 ^ab^	3.97 ± 0.78 ^a^	**	ns
	SFO	7.73 ± 1.77 ^a^	5.44 ± 0.70 ^b^	5.75 ± 1.16 ^b^	ns	4.93 ± 0.38 ^b^	5.32 ± 0.68 ^ab^	5.36 ± 0.57 ^ab^	6.75 ± 0.96 ^a^	**	ns

Data were presented as a mean value of 12 replicates for storage time and 9 for treatment. Cyanidin 3-*O-*glucoside (C3G), Cyanidin 3-*O-*rutinoside (C3R), Peonidin 3-*O-*glucoside (P3G), Peonidin 3-*O-*rutinoside (P3R), Sum of anthocyanins (SAN), Neochlorogenic acid (NEO), *p*-Coumaroylquinic acid (PCQ), Chlorogenic acid (CHL), *p*-Coumaric acid (PCO), Sum of hydroxycinnamic acids (SHA), Quercitin 3-*O-*rutinoside (Q3R), Kaempferol 3-*O-*rutinoside (K3R), Isoharm 3-*O-*rutinoside (I3R), Sum of flavonols (SFL), Procyanidin PB1 (PB1), Procyanidin PB2 (PB2), (+)-Catechin (CAT), (−)-Epicatechin (EPI), Sum of flavan-3-ols (SFO).^1^ In each row by storage time and treatment, different letters indicate a significant difference at *p* ≤ 0.05 (Tukey’s test). *p*^#^ values for storage time: ****: *p* ≤ 0.0001; ns: not significant. *p*^##^ values for treatment: ***: *p* ≤ 0.001; **: *p* ≤ 0.01; *: *p* ≤ 0.05; +: *p* ≤ 0.1; ns: not significant. *p*^###^ values for storage time × treatment interaction: ***: *p* ≤ 0.001; *: *p* ≤ 0.05; +: *p* ≤ 0.1; ns: not significant.

## Data Availability

No new data were created or analyzed in this study. Data sharing is not applicable to this article.
